# Biotic interactions biogeography: A framework for understanding how species interactions shape biodiversity patterns across scales

**DOI:** 10.1371/journal.pbio.3003813

**Published:** 2026-06-15

**Authors:** Nuria Galiana, Miguel B. Araújo

**Affiliations:** 1 Department of Biogeography and Global Change, National Museum of Natural Sciences, Madrid, Spain; 2 Mediterranean Institute for Agriculture, Environment and Development & CHANGE – Global Change and Sustainability Institute, University of Évora, Évora, Portugal; 3 German Centre for Integrative Biodiversity Research (iDiv), Halle-Jena-Leipzig, Leipzig, Germany

## Abstract

The integration between biogeography and ecology has been historically limited due to the lack of data on biotic interactions across large spatial scales. The emergence of new methods and high-quality ecological network data at biogeographical scales are paving the way for a deeper integration of biogeography and ecology. This Essay examines this integration through three interconnected research areas: the effects of biotic interactions on species distributions; the influence of environmental gradients on biotic interactions; and the effects of biotic interactions on the environment. Recent progress and primary challenges are discussed, and suggestions provided on how to advance understanding of biodiversity patterns and processes across scales.

## Abstract 2

Biogeography and ecology have long developed in parallel. This Essay explores how new data on species interactions across large scales now allow their integration, opening fresh insights into how biodiversity is shaped (and reshaped) across Earth.

## Introduction

Biogeography and ecological research have traditionally developed along parallel tracks, with their full integration hindered by limited data on species interactions across large spatial scales [1–3]. While biogeography has focused primarily on broad-scale biodiversity patterns, community ecology has traditionally studied species interactions and community structure at smaller scales. This partial disconnect has constrained our ability to predict ecosystems’ responses to global environmental change.

The historical gap in understanding of biotic interactions (Box 1) at biogeographical scales, often termed the ‘Eltonian shortfall’ [1], refers to the “scarcity of knowledge about intra- and inter-specific interactions, responses of species to environment and the effects of species on ecosystems” [2,3]. Many fundamental concepts in community and ecosystems ecology, such as the distinction between fundamental and realized niches (Box 1), latitudinal gradients in interaction strength, and assembly rules, inherently bridge biogeography and species interactions. However, these concepts and intuitions were often difficult to test due to limited data on biotic interactions across large spatial extents.

**Box 1. Glossary.**

**Biotic interactions**

Relationships between organisms in which they affect one another’s fitness, survival, or population dynamics, including predation, competition, mutualism, commensalism, and amensalism.

**Niche**

The ecological role and requirements of a species within its environment. The Hutchinsonian niche refers to the n-dimensional hypervolume of environmental conditions where a species can survive and reproduce, whereas the Eltonian niche refers to the functional role of a species in the community, including its trophic position, resource use, and ecological interactions.

**Ecological networks**

Representations of biotic interactions within an ecosystem where species are depicted as nodes and their interactions as links, allowing for the analysis of community structure and dynamics.

**Trophic cascade**

Indirect effects that propagate across trophic levels through chains of interactions. In top-down cascades, predators suppress herbivore populations, indirectly benefiting primary producers; in bottom-up cascades, changes in nutrient availability or primary productivity propagate upward through consumers. These cascading effects can alter the structure and function of ecosystems across spatial scales.

**Species distribution models (SDMs)**

Computational tools that combine observations of a species occurrence or abundance with environmental data to predict species’ geographic distributions based on their environmental requirements.

**Joint species distribution models (JSDMs)**

Extensions of SDMs that simultaneously model multiple species’ responses to environmental predictors while accounting for residual correlations between species.

**Metawebs**

Potential networks of biotic interactions that represent potentially feasible interactions between species in a regional pool, regardless of whether all species interact locally.

**Trophic theory of island biogeography (TTIB)**

An extension of MacArthur and Wilson’s theory of island biogeography that incorporates trophic constraints, recognizing that consumers can only colonize islands where their prey species are present.

**Fig 1:**
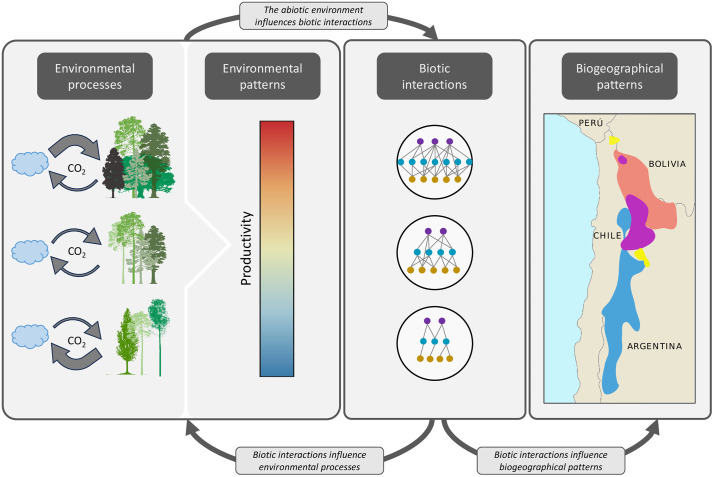
Biotic interactions biogeography. The abiotic environment influences how species interact and the structure of the ecological communities. Several sources of environmental variation can affect biotic interactions, such as climatic conditions or habitat isolation. In turn, biotic interactions strongly influence environmental processes through, for instance, the modification of physical landscapes, their influence on nutrient cycling and soil formation, or their impact on the carbon cycle. Biotic interactions also influence large-scale biodiversity patterns. A classic example is the distribution of *Liolaemus* lizards in South America, where species minimize competition through niche differentiation and specialization, allowing for the coexistence of multiple species within the same or nearby habitats [21]. Colors in the right panel correspond *to Liolaemus andinus* clade in blue, *Liolaemus erguetae* clade in purple, *Liolaemus poecilochromus* clade in yellow and *Liolaemus multicolor* clade in brick red.

Over the past decade, considerable progress has been made in addressing Eltonian data and knowledge gaps [4,5]. The emergence of new methodologies for data collection [6] and for reconstructing ecological networks across large spatial scales [7] has paved the way for a deeper integration of biogeography and ecology, enabling researchers to investigate questions that span traditional disciplinary boundaries [8]. Recent studies have also demonstrated the value of integrating biogeography and ecology. Environmental factors such as climate and anthropogenic pressure have been shown to determine the trophic organization of ecological communities worldwide [9–12]. Concurrently, biotic interactions are increasingly being recognized as influencing biogeographical patterns, including species’ geographical ranges [13], abundance distributions, and diversity patterns [14]. Importantly, these interactions are not static across space: a species may change its interactions depending on its location within its range, with potential consequences for local community structure and broader biogeographical patterns [15–17]. Studies have also revealed that biotic interactions can mediate species responses to environmental changes and perturbations [18,19], effectively modifying how organisms experience abiotic conditions [20] (Fig 1).

Biotic interactions biogeography emerges at the intersection of these fields, focusing on reciprocal effects between species interactions and biogeographical patterns (Box 2). In this Essay, we examine this integration through three interconnected research areas: the effects of biotic interactions on species distributions; the influence of environmental gradients on biotic interactions; and the effects of biotic interactions on the environment. For each area, we highlight recent advances and key challenges, providing suggestions on how to advance understanding of biodiversity patterns across scales.

**Box 2. Foundations of biotic interactions biogeography.**

Biogeography is the study of the distribution of life across space and time. It seeks to decode the patterns and dynamics of biodiversity, typically studied through examination of species richness, distributions, or associated properties such as range sizes. With its roots tracing back to the early 1800s, the field of biogeography has a rich history [22,23]. Despite its long-standing tradition, the integration of biotic interactions into biogeographical analysis has traditionally been limited, underscoring the focus on abiotic factors rather than on the complex interplay between species.

By contrast, ecology investigates the relationships between organisms and their environment across multiple scales of biological organization, from populations to ecosystems. It encompasses population dynamics, species interactions, community structure, ecosystem processes, and their reciprocal effects on the physical environment. Within community ecology, tools from network research have enabled numerous theoretical and empirical studies that have identified general patterns and mechanisms of species interactions and the structure of the complex networks they form across various systems (reviewed in [12,24–26]). These patterns impact community dynamics, strongly influencing ecosystem stability and resilience to disturbances [25,27,28]. They also underpin essential ecosystem functions such as primary production, nutrient cycling, pest control, or pollination [29,30]. Analyzing ecological communities as networks of biotic interactions has been crucial for understanding biodiversity organization within communities and for predicting their responses to environmental changes. However, these studies have been traditionally focused on local scales, dismissing the influence of biogeographical components in determining the structure and functioning of ecological communities, though notable efforts have been made to bridge this divide [5,8,31–34].

Biotic interactions biogeography emerges from the integration of biogeography with community and ecosystem ecology*,* and it focuses on the reciprocal effects of biotic interactions and biogeographical patterns. It studies how environmental factors affect biotic interactions and, in turn, how these interactions influence the mechanisms governing the distribution of structural and functional facets of life across Earth’s major environmental gradients. The term ‘environment’ encompasses not only abiotic processes affecting the planet’s surface but also the socioecological dynamics of geological significance—traditionally recognized as the driving forces of the anthropocene.

## The effect of biotic interactions on distributional patterns

Biotic interactions have traditionally been considered to influence local communities, while having limited broader-scale effects on species diversity [35] and distributions [36]. However, recent research has begun to demonstrate their influence on broad-scale distributional patterns through mechanisms such as predation [37], parasitism [38], or facilitation [39]. Yet the extent to which biotic interactions systematically shape large-scale distribution patterns remains empirically underexplored, conceptually under-integrated, and methodologically challenging to quantify, highlighting a fundamental gap in biogeographical research.

### Broad scale signatures of biotic interactions on species distributions

Simulations suggest that the effects of biotic interactions at biogeographical scales are likely stronger when interactions involve strong dependencies [40]. Empirical evidence supports this view: predators influence the abundance, distribution, and range limits of prey species in terrestrial, freshwater, and marine systems [14,37,41]. Similarly, keystone plants can exert broad-scale effects on the distribution and diversity of frugivorous birds [42].

Experimental studies further reinforce the importance of species interactions in determining range limits and distributional dynamics. For example, the establishment and persistence of alpine plants in novel climates are constrained not only by climate but also by the presence of competitors [43]. In another study, upslope experimental translocations showed that when lowland herbivorous insects colonize higher altitudes, their feeding behavior alters plant community structure [44]. By modifying plant height, these insects create conditions that enhance plant species coexistence, especially benefiting smaller plants. In line with these findings, mammalian herbivores drive low-elevation range limits of alpine plants; their upward expansion could trigger local extinctions by suppressing plant population growth [45]. These and other studies suggest that top-down regulation by herbivores and carnivores can have a disproportionately important role in constraining redistributions of species and trophic cascades (Box 1) across ecosystems [46,47]. Yet, despite growing evidence for the role of biotic interactions in constraining species distributions, these processes remain insufficiently incorporated into biogeographical models, highlighting a persistent disconnect between local ecological mechanisms and large-scale predictive frameworks.

### Biotic interactions as a knowledge gap

Although the influence of biotic interactions is increasingly being recognized, biogeographical studies continue to prioritize climate (as a first-order driver of species distributions through its control over physiological tolerance limits) and historical processes that shape initial range conditions and constrain movement [1]. The role of biotic interactions is often underexplored, typically reduced to the most immediate effects on focal species [38] rather than the broader network of ecological relationships in which they are embedded.

Yet ecological communities are structured by a web of direct and indirect interactions, the complexity of which can give rise to emergent behaviors that are not predictable from pairwise relationships alone [48,49]. Capturing these complex community-level dynamics is essential for understanding how interactions influence large-scale biodiversity patterns. Incorporating ecological networks can substantially improve our ability to characterize current species distributions and anticipate their reorganization under global change. However, realizing this potential requires modelling approaches that can formally integrate biotic interactions alongside environmental drivers in species distribution frameworks (Box 3).

**Box 3. Network inference and global datasets: bridging scales and methodological challenges.**

Empirically documenting ecological networks at biogeographical scales presents significant challenges of data collection and harmonization [50]. These include inconsistent sampling methodologies across studies, uneven geographical coverage, and taxonomic standardization issues that are often overlooked. To address these limitations, researchers have developed approaches for studying species interactions across large spatial extents.

The use of potential networks of biotic interactions, termed ‘metawebs’ [24] or ‘backbone networks’ [7], has become essential for analyzing ecological networks at biogeographical scales [5]. These represent potential interactions that could occur given species co-occurrence, regardless of whether all are realized in any specific local community. An important consideration here is that the interaction data used to construct metawebs often derive from a limited set of regions, and interaction rules documented in one biome may not transfer reliably to another, introducing an additional source of uncertainty when applying metawebs beyond their region of origin.

The relationship between potential interactions (metawebs) and realized interactions (locally observed networks) reflects a central challenge in biotic interactions biogeography. Local networks represent a subset of the regional metaweb, filtered by environmental conditions affecting species distributions and phenologies, context-dependent interaction strengths that vary with abiotic conditions [51,52], local adaptation in interaction-relevant traits [53], and the presence/absence of third-party species that modify interactions [54,55]. Current applications of metawebs in biogeographical studies typically assume relatively fixed interaction rules across environmental gradients. The field requires methods that account for the dynamic nature of interactions across space and time. Future advances might involve techniques for estimating interaction strength variation with environmental conditions [51,56], approaches for modelling interaction rewiring [54], and frameworks for integrating abundance-based interaction probabilities into metawebs. Validation through targeted field sampling will be crucial for understanding extrapolation limits [57].

Beyond new approaches to infer ecological networks, advances in biotic interactions biogeography will also require improved data on biotic interactions across environmental gradients. Recent years have seen impressive efforts to create standardized global databases that unify independently sampled networks. Examples include Mangal [58], Web of Life, GloBI, RecrutiNet [59], and Fungal databases, which provide standardized ecological networks with relevant metadata and tools for analysis. These databases enhance comparability but face challenges of sampling bias [60,61], inconsistent methodologies, and uneven geographical coverage [4]. Taxonomic harmonization across datasets represents a further methodological challenge, as incongruent nomenclature can lead to overestimation of species and interaction diversity when aggregating local networks. Potential approaches to mitigate these limitations include incorporating sampling effort and methodology as covariates in comparative models, and developing frameworks that explicitly account for sampling bias in the inference of interaction networks. The integration of standardized network databases with environmental data layers represents a promising avenue for addressing questions at the intersection of biogeography and species interactions [12].

### Integrating biotic interactions into distributional models

The species distribution modelling literature exemplifies the evolving role of biotic interactions in biogeographical research. Initially centered on abiotic predictors, species distribution models (SDMs; Box 1) have gradually incorporated biotic interactions as covariates to better capture the complexity of species distributions [14,38]. This shift has proven valuable; for example, both biotic and abiotic variables each independently explained around 20% of variation in alpine tree distributions [62]. The importance of accounting for interactions becomes even more evident under climate change scenarios. For example, trophic interactions can slow the pace of range shifts, especially in larger-bodied species, and help maintain historical community structure [63]. Nonetheless, although some studies have sought to mechanistically integrate biotic interactions into climate-driven models [64], empirical evidence for consistent improvements in the predictive accuracy of more complex mechanistic or hybrid models remains scarce [65].

Joint species distribution models (JSDMs; Box 1) offer a phenomenological framework for examining multiple species’ responses to environmental predictors simultaneously [66,67]. By estimating residual covariation in species distributions after controlling for environmental effects, JSDMs aim to capture potential signals of biotic interactions. However, this residual covariance structure has been widely debated and shown to be insufficient for inferring true biotic interactions [68]. Residual correlations can emerge from multiple sources, such as unmeasured environmental covariates, sampling biases, or stochastic variation, and do not reflect ecological mechanisms per se [69]. Therefore, while JSDMs represent a methodological advance for modelling species assemblages, particularly suited to modelling rare species when data are sparse [70], they remain limited in their capacity to infer the complex web of biotic interactions that govern species distributions [71].

To move beyond phenomenological approaches that rely on residual patterns, recent efforts have sought to explicitly incorporate ecological knowledge (such as known interspecific interactions) into species distribution modelling. ELGRIN provides such a framework by using a Markov random field to integrate empirical metawebs (Box 1) alongside environmental predictors, thereby jointly modelling abiotic and biotic components of community structure [72]. This allows for a more transparent assessment of how interactions, in aggregate, shape species distributions across landscapes. Other approaches have proposed modifying JSDMs to incorporate network-derived information, either by applying them to subsets of species known to interact within specific regions or by including interaction-based covariates in SDMs for focal species. However, these hybrid strategies remain methodologically underdeveloped, and defining ecologically meaningful species subsets or retaining the joint modelling structure of JSDMs poses significant challenges.

### Biotic interactions and co-occurrences

The spatial co-occurrence of species across landscapes is partly shaped by their biotic interactions. Co-occurrence patterns can therefore serve as a valuable source of information about those interactions, making the reciprocal relationship between co-occurrence and biotic interactions a critical entry point for linking ecological and biogeographical processes. When direct data on species interactions are lacking, one strategy is to infer potential associations from observed co-occurrences [73,74]. While co-occurrence alone cannot reliably identify specific pairwise interactions [75] owing to confounding factors such as shared habitat preferences, environmental tolerances, or abundance and neutral effects, recent work has shown that co-occurrence network patterns can provide valuable information about community structure and assembly processes [76]. For example, although ~20% of co-occurring species truly interact, the structure of co-occurrence networks nonetheless reflects underlying interaction processes. In particular, generalist species—those that interact with many other species—co-occur in space disproportionately with other species relative to specialists, indicating that spatial associations are filtered by the same ecological processes that govern who interacts with whom. This signal can be detected by comparing how interaction partners are distributed across species in co-occurrence versus interaction networks [76].

Understanding reciprocal feedbacks (e.g., how interactions generate co-occurrence patterns, and how those patterns can, in turn, inform interaction inference) is critical for advancing network ecology and spatial modelling. Embedding co-occurrence analyses within network frameworks may thus help identify key structural roles (e.g., keystone species or vulnerable nodes) [77], and offer insights into how communities reorganize under environmental change [78]. Caution is nonetheless warranted given that the realization of interactions not only depends on co-occurrence but also on local conditions, population densities, and phenological overlap [54].

Beyond co-occurrence, approaches that incorporate demographic information offer a more direct route to capturing ecologically meaningful interactions. Recruitment networks, for instance, infer positive or negative effects on plant establishment by comparing recruitment success near canopy individuals to that in open spaces, providing interaction estimates grounded in demographic consequences rather than spatial association alone [79]. Such approaches move closer to capturing the fitness consequences of interactions and point towards a more demographically explicit understanding of how biotic interactions shape species distributions across landscapes [80].

### Challenges and perspectives

Despite growing recognition of biotic interactions in shaping species distributions, key challenges remain. Local interaction data are often mismatched in scale with the broad biogeographical patterns they aim to explain [40]. Additionally, disentangling the effects of biotic interactions from correlated environmental factors is difficult, as interacting species often share similar niches [69]. Interaction strength also varies across environmental gradients [51,52], making static representations inadequate.

Progress will depend on expanding standardized interaction databases [4,58] and conducting targeted field experiments across environmental gradients [57]. Process-based models that integrate demographic mechanisms offer promise both for scaling local interactions to regional patterns and for moving beyond interaction occurrence towards fitness-based estimates of interaction strength, ultimately improving predictions under global change [65].

## The effect of environmental gradients on biotic interactions

Environmental conditions affect the distribution of species, but also the biotic interactions among them. A widespread view is that biotic interactions are more specialized at lower latitudes owing to a more benign and constant climate [81,82]. However, evidence for this pattern is mixed, with methodological inconsistencies and limited replication across environmental gradients obscuring clear conclusions. Despite important contributions from distributed experiments and collaborative networks, large-scale patterns have often been pieced together from different data sources using varying methodologies, with insufficient standardized replication to establish robust generalities. Yet, this knowledge is crucial to predict how ecological communities respond to environmental changes.

### Latitudinal gradients

There are several sources of environmental variation that can affect biotic interactions and the structure of ecological communities. A prominent one is climate. Climate can significantly influence biotic interactions by altering their strength [51,52], modifying biomass distribution across trophic levels [83–85], and determining net primary productivity, which correlates with sharp transitions in terrestrial trophic communities [10,11]. While several studies have characterized geographical variation in interaction networks over the past decade (see [12,50,86] for reviews), consensus remains elusive. For instance, some studies showed an increase in network specialization and predation risk towards the tropics [52,87], while others found the opposite pattern [88]. Interestingly, these patterns depend on factors such as the hemisphere analyzed [89], the measure of biotic specialization used [53], and the spatial scale under consideration [90], underscoring the importance of developing a mechanistic understanding of the observed patterns and working with standardized data.

A novel approach to standardizing data on biotic interactions across large environmental gradients is the use of metawebs (Box 3). Metawebs analyze variation in community structure at large spatial scales, and their use for the analyses of potential interaction network variation across environmental gradients is becoming standard practice [10,11,33,91,92]. In marine systems, for instance, network structure correlates with sea surface temperature at the global scale, with communities being more diverse and complex towards the tropics [91]. Similarly, in the Barents Sea, water temperature and sea ice were the major determinants of variation in the structure of food webs [92]. In terrestrial systems, community trophic structures of mammals and birds closely match the major biomes on earth [11], with the simplest trophic structures found in the most depauperated regions [93].

**Fig 2:**
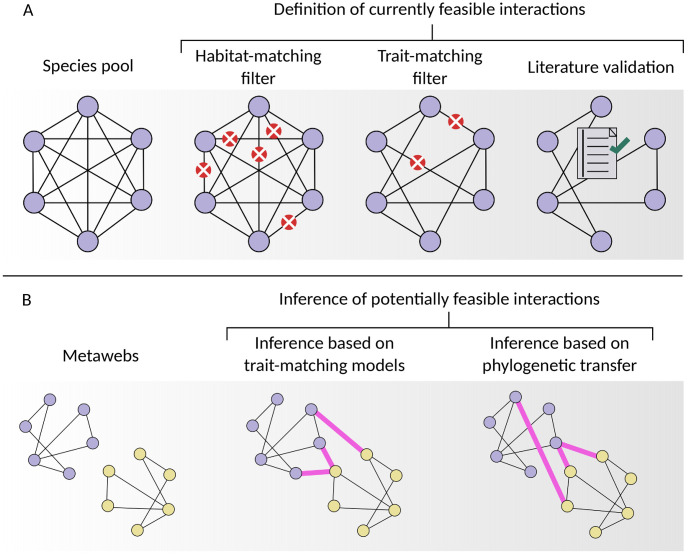
Schematic representation of metaweb construction approaches. **A.** Definition of currently feasible interactions among species that currently co-occur. Starting from a regional species pool in which pairwise interactions across the full species pool are considered as candidates, feasible interactions are progressively identified through a series of filters: a habitat-matching filter removes interactions between species that do not co-occur in the same habitat (red crossed links); a trait-matching filter further removes interactions that are incompatible given the functional traits of the species involved, identifying forbidden links (pairwise interactions between co-occurring species that cannot be realized due to biological incompatibilities); and literature validation confirms empirically documented interactions, producing a data-driven metaweb of currently plausible interactions. **B**. Inference of potentially feasible interactions. Model-based approaches extend metaweb construction beyond currently co-occurring species, allowing inference of interactions between species that do not presently co-occur but may do so under future conditions, for instance as a result of climate-driven range shifts. Two such approaches are illustrated: inference based on trait-matching models, which predict interactions from functional trait combinations, and inference based on phylogenetic transfer, which extrapolates interaction rules from closely related species. Inferred interactions are shown as pink links added to the existing metaweb structure. Together, panels A and B illustrate the continuum between metawebs anchored in current co-occurrence and metawebs that incorporate future or otherwise non-co-occurring species pairs.

When considering metawebs, it is important to recognize that metaweb construction methods lie along a continuum between two conceptual extremes. At one end, metawebs can be assembled by aggregating empirically documented interactions from local networks across a region, producing a data-driven compilation of observed interactions. At the other end, metawebs can be inferred using model-based approaches (including trait matching, phylogenetic relatedness, and co-occurrence patterns [7,94–96]) that extrapolate beyond directly sampled interactions to predict potential links across the full regional species pool. These two approaches differ not only in their data requirements but also in what they capture: empirically aggregated metawebs are constrained by sampling effort and geographical coverage, whereas model-based metawebs offer broader taxonomic and spatial coverage at the cost of greater uncertainty. In practice, robust metawebs often combine both approaches [91,97–99] (Fig 2), but awareness of this distinction is essential when interpreting results or comparing metawebs across studies.

An important caveat is that metaweb-based analyses typically capture environmentally driven variation in network structure indirectly, through changes in which species co-occur locally, rather than through changes in interaction rules themselves. When interaction rules are derived from phylogeny or fixed traits, the spatial signal in the realised network reflects species turnover filtered through a static set of potential interactions. This limitation is particularly relevant given growing empirical evidence that trait matching itself varies geographically: global analyses of avian frugivory networks have reported either stronger trait matching towards the tropics, shaped by climate and biogeographic history [100], or the opposite pattern, with weaker matching in the tropics [101], though both studies converge in finding weaker trait matching on islands and in depauperate regions. This is a meaningful limitation when the goal is to understand how environmental conditions modify interactions per se—for instance, by altering interaction strength, rewiring partners, or shifting context-dependent outcomes—rather than community composition (see Box 3).

### Environmental isolation

**Fig 3:**
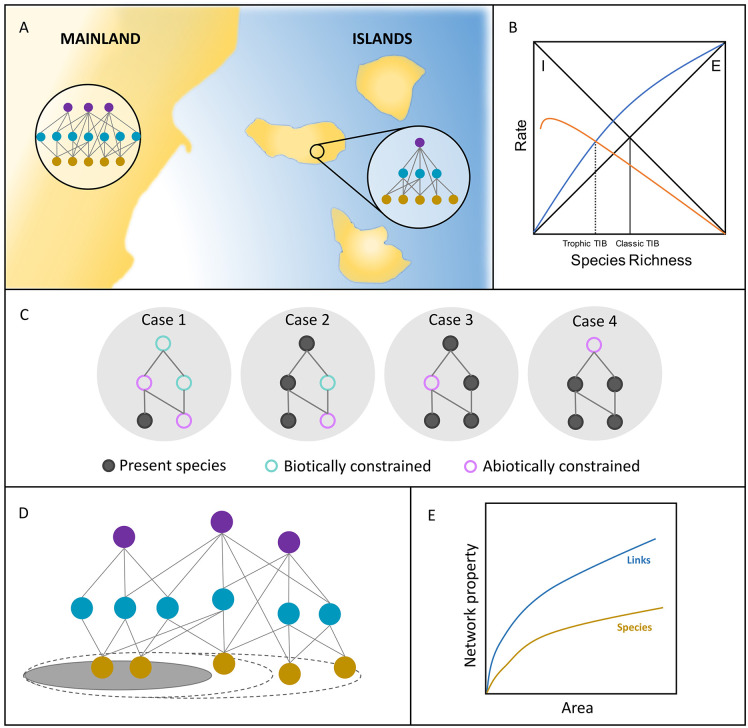
Island biogeography and the spatial scaling of ecological networks. **A.** Comparison between network structures on the mainland and on islands. **B**. Trophic theory of island biogeography adapted from [107]. Black lines represent the classic theory of island biogeography (TIB) and colored lines represent the trophic theory of island biogeography (TTIB). In both cases the diversity equilibrium is reached when immigration rate is equal to extinction rate (intersection between I and E). **C**. Four scenarios illustrating how abiotic constraints at lower trophic levels can generate biotic constraints at higher ones, depending on prey availability. Case 1: Two species are abiotically constrained (one basal, one intermediate), preventing the top predator from persisting due to a trophic constraint. Case 2: One basal species is abiotically constrained, causing its sole predator to be biotically constrained. Case 3: One intermediate species is abiotically constrained, but because the top predator has an alternative prey, no biotic constraint propagates upward. Case 4: Only the top species is abiotically constrained, with no trophic cascading effect on other species. **D**. Illustration of the spatial scaling of network structure. **E**. Schematic representation of the changes in network properties as the size of the area of observation increases.

The effects of ecosystems being depauperated on ecological communities have been extensively studied through habitat fragmentation and isolation [27,102,103]. Habitat fragmentation simplifies communities and reduces their robustness to coextinctions [102], whereas isolation particularly affects large-bodied consumer species, disturbing community structure and functioning [27,103]. On islands, environmental isolation also influences biotic interactions following long-recognized trends in species richness (Fig 3A). Island pollination networks, for instance, have fewer interacting species and more simplified structures than their mainland counterparts [87,104]. Species-poor insect communities of islands promote the establishment of highly generalized species [105], which further influences other commonly found patterns in networks, such as interaction asymmetry and nestedness [104,106].

These island patterns have been theoretically explored by Gravel and colleagues [107] through the trophic theory of island biogeography (TTIB; Box 1), which extends the classic theory of island biogeography [108] by incorporating species interactions. The TTIB includes a trophic constraint: species can only colonize and persist if at least one of its prey is present. This constraint modifies the classic diversity equilibrium, slowing species accumulation and favoring early establishment of generalist species (Fig 3B). Importantly, trophic constraints at higher trophic levels can arise indirectly from abiotic constraints acting on lower ones: when abiotic conditions exclude a prey species, its predators may face biotic constraints even in otherwise suitable environments. Whether such constraints propagate upward depends on prey availability; predators with alternative prey may persist despite the loss of one prey species, whereas more specialized consumers are more vulnerable to cascading exclusion (Fig 3C). TTIB predicts that network structure varies with island size, as larger areas support sufficient diversity for all consumers (not only generalists) to find prey, whereas smaller areas select for generalist species. Recent theoretical and empirical work has demonstrated that area size affects both species richness and broader aspects of community structure (Fig 3D–3E) [109,110].

### Challenges and perspectives

Despite the remarkable advances in understanding the effects of different environmental drivers on biotic interactions, we are still far from a thorough comprehension of how ecological communities vary across environmental gradients. A major challenge is the definition of meaningful boundaries [106]. Local communities are often analyzed as independent entities, yet they are spatially interconnected [12,111]. Notable progress has nonetheless been made in this area, with recent work showing that large-scale ecological boundaries (such as ecoregions and biomes) can drive abrupt spatial discontinuities in interaction structure [112]. Analogous challenges arise in the temporal dimension, where a given snapshot in time is not independent from the rest of the temporal dynamics of the system. Identifying meaningful boundaries is thus fundamental for interpreting and comparing the patterns observed. The integration of metacommunity concepts [113] with detailed interaction network analyses across environmental gradients represents a promising area of research that could help address boundary definition challenges.

Parallel to the artificial delimitation of local communities in space and time, studies tend to focus on a single interaction type (i.e., antagonistic or mutualistic). Yet communities involve multiple interaction types that are interconnected through shared species: a plant, for instance, may simultaneously be a resource in a trophic network and a mutualistic partner in a pollination network, meaning that in reality these interaction types form parts of a single, integrated ecological web that is partitioned for analytical convenience [55,114]. A promising framework for moving beyond this limitation is the use of multilayer networks [115], where different interaction types are represented as distinct but interconnected layers linked through shared species. The combination of multiple interaction types affects different aspects of community stability and the response to environmental perturbations [116–118]. Moreover, different interaction types may exhibit distinct responses to environmental gradients, potentially varying in both their sensitivity and direction of response [119]. Understanding the response of ecological communities to future environmental change will, therefore, benefit from examining multiple interaction types simultaneously [110].

The integration of space, time, and interaction types within ecological networks requires appropriate sampling. It is indeed challenging to achieve sufficient sample sizes to fully characterize species interactions, particularly when including species abundances and interaction strengths [57]. Sampling completeness and bias significantly impact the estimation of community structure [60, 61], hindering understanding of how environmental gradients affect biotic interactions and ecological communities. Environmental DNA sampling [6], automated camera trap networks [120], and emerging techniques to infer biotic interactions (Box 3) can collectively contribute to alleviating these sampling limitations, providing complementary approaches to document species interactions with greater standardization and spatial coverage across environmental gradients.

## The effect of biotic interactions on the environment

Parallel to the growing recognition that biotic interactions affect large-scale biodiversity patterns, there is also acknowledgment of the role that biotic interactions have in shaping the very environmental conditions that underpin those patterns [41,121]. Jones and colleagues first defined ecosystem engineers as organisms that directly or indirectly modulate the availability of resources to other species by causing physical state changes in biotic or abiotic materials [121]. These ecosystem engineering effects exemplify how biotic interactions transcend traditional ecological boundaries, simultaneously influencing biodiversity patterns, ecosystem processes, and the physical environment itself.

### Physical landscape modification

A classic example of the influence of biotic interactions on entire ecosystems is the reintroduction of wolves to Yellowstone National Park in the USA, where trophic cascades affecting elk populations created landscape mosaics of forests, grazed prairies, and disturbed habitats, restoring ecosystem functioning [37]. These interactions reshaped physical environments, creating heterogeneity in landscape structure.

In aquatic ecosystems, biotic interactions have a profound impact on water quality by regulating nutrient levels, controlling organic matter breakdown, and influencing the physical and chemical properties. A classic illustration is the trophic cascade in lakes, where piscivorous fish suppress planktivorous fish, which in turn releases zooplankton populations that graze on phytoplankton. The presence of top predators thus indirectly limits algal biomass and helps maintain water clarity, whereas their loss can contribute to eutrophication and oxygen depletion [122]. Additionally, the presence or absence of key species, such as filter feeders (e.g., mussels), can directly influence water clarity and nutrient levels by filtering particles and cycling nutrients through the ecosystem [123].

### Biogeochemical cycling

In terrestrial ecosystems, biotic interactions substantially contribute to soil formation through both physical and chemical means, and influence the cycling of key nutrients such as nitrogen and phosphorus. For example, mycorrhizal fungi form symbiotic relationships with plant roots, aiding in nutrient absorption and contributing to soil structure and water retention, ultimately promoting plant growth and ecosystem stability [124]. Similarly, nitrogen-fixing bacteria, such as Rhizobium, form symbiotic relationships with leguminous plants. These bacteria convert atmospheric nitrogen (N₂) into ammonia (NH₃), a form of nitrogen usable by plants, thereby enriching soil nitrogen levels. This process is essential for nitrogen cycling, as it makes atmospheric nitrogen accessible to plants, a key nutrient that is often a limiting factor in ecosystems [125,126].

Soil organisms such as fungi and bacteria are not only essential for the cycling of key nutrients such as nitrogen and phosphorus, but also have a crucial role in the global carbon cycle. By decomposing dead organic matter, they release carbon dioxide (CO₂) into the atmosphere, affecting both soil carbon storage and atmospheric CO₂ levels [127]. Besides these direct biota-mediated effects on carbon cycling, biotic interactions between producers, consumers, and decomposers can induce important changes in the rate of carbon sequestration. For instance, herbivores can, on the one hand, reduce vegetation cover, limiting the carbon sequestration potential of these ecosystems. On the other hand, they also influence soil processes through physical disturbances such as trampling, which can alter root growth, leading to an increase in carbon storage [128]. Predators, in turn, can exert top-down control over herbivore populations and indirectly support higher plant biomass and greater carbon uptake [129,130]. Thus, biota-mediated effects on carbon cycling occur through a diverse array of biotic interactions that operate simultaneously, and the balance and structure of these complex food webs qualitatively change the dominant control over carbon cycling [130–132].

Importantly, biotic interactions can affect carbon cycling across different spatial scales—from micrometer to continental scales—depending on the organisms involved [132]. At the micro-environment scale, the strong biotic interactions between plants, soil fauna, and microbes occurring at the rhizosphere generate a hotspot for belowground carbon turnover [132,133]. At larger spatial scales, carbon can be exchanged across ecosystems through the biotic interactions among animals that move along large ranges. For instance, seed consumption by migratory birds promotes long-distance plant dispersal, which can influence vegetation composition and structure at the destination sites, with downstream consequences for biomass accumulation and ecosystem carbon storage [134].

### Climate regulation

Beyond carbon emissions, biotic interactions also influence microclimates and broader climate patterns. Vegetation communities, shaped by competitive, facilitative, and trophic interactions among species, have a critical role in regulating temperature, humidity, and wind patterns at both local and regional scales [135,136]. For example, the competitive and facilitative interactions between tree species in a forest create multi-layered canopy structures that collectively alter atmospheric conditions by shading the ground, reducing surface temperatures, and increasing humidity through transpiration. The specific composition of tree communities, shaped by these biotic interactions, determines the magnitude of these effects on local microclimates, with consequences for species distributions under global change [136–138].

Additionally, trophic interactions between plants and their herbivores or pathogens directly modify vegetation structure and composition, which in turn influences the reflectivity (albedo) of the surface, contributing to temperature regulation [136,137]. Similarly, when insect outbreaks or pathogen spread preferentially affect certain tree species due to host-specific interactions, the resulting changes in forest community composition can alter hydrological cycles. These interaction-driven changes in forest communities can reduce rainfall patterns, as altered forest structures cycle different amounts of water vapor into the atmosphere through transpiration, affecting cloud formation and precipitation regimes [139,140].

These biotic-mediated changes in vegetation composition and land surface properties also influence fire regimes [141,142]. In savannas and grasslands, grazing by herbivores can reduce fuel loads, lowering fire frequency. Conversely, insect-induced tree mortality can create large amounts of dead wood, which significantly increases the likelihood of fires. Fires, in turn, release large amounts of CO₂ and aerosols into the atmosphere, affecting both local and regional climatic regimes [142]. These examples illustrate how species interactions cascade to influence climate processes across multiple spatial scales.

### Challenges and perspectives

The interplay between organisms and the environment is mediated by the strength and balance of all the biotic interactions involved, which shape how ecosystems respond to and influence climatic changes. Comprehensively accounting for these multilevel, spatially dynamic effects across landscapes presents several key challenges. Scale mismatches between fine-resolution interactions and broader environmental processes, the strong context-dependency of biotic effects on ecosystem function, and the long temporal scales over which many feedback mechanisms operate all complicate our ability to develop robust predictive frameworks.

Despite these challenges, emerging approaches offer promising paths forward. New geospatial statistical methods, combined with remote-sensing data and spatial ecosystem modelling, are enhancing our understanding of how biotic-mediated processes regulate environmental patterns at global scales [129,131]. Integrating interaction network data with environmental monitoring, and developing more sophisticated modelling approaches that explicitly incorporate spatially and temporally variable interaction strengths, interaction rewiring under environmental change, and indirect effects across trophic levels will be the next essential steps. These developments will be critical for anticipating how changes in species interactions under global change will alter carbon cycling, microclimate regulation, and other ecosystem processes with direct consequences for conservation and management in a rapidly changing world.

## Conclusions and future directions

Biotic interactions biogeography strengthens the links between biogeography and ecology by integrating the study of biotic interactions with the analysis of species distribution patterns and community or ecosystem dynamics. Often treated as separate, recent advances in data collection, network analysis, and modelling provide the tools necessary to integrate both abiotic and biotic factors in the analysis of biogeographical patterns and processes. This integrative approach uniquely positions the fields of biogeography and ecology to link patterns and processes across scales.

Looking forward, several critical challenges will require focused scientific attention. First, methodological advances must address the complexity of ecological interactions. Developing standardized metrics of interaction strength that are comparable across environmental gradients and interaction types remains a fundamental need. Similarly, integrating multiple interaction types (mutualistic, antagonistic, and facilitative) into unified network models will be essential for capturing the full complexity of ecological communities. Detecting and quantifying higher-order interactions and emergent indirect effects that manifest at biogeographical scales represents another frontier requiring novel analytical approaches. Such effects may propagate across large spatial and temporal scales through species with broad ranges, migratory behavior, or long lifespans, the interactions of which can connect otherwise distant or temporally separate communities [134].

Second, climate change presents unprecedented challenges for understanding interaction dynamics. As novel community compositions emerge under changing climates, fundamental questions arise about how species interaction patterns will shift and whether invasive species and their novel interactions will reshape future biodiversity patterns. Trait-based frameworks offer a promising avenue for addressing these questions, as functional traits can help predict how climate change will alter spatial and temporal mismatches between interacting species, trigger novel interactions, and drive secondary extinctions [143]. A critical consideration for predictive frameworks is whether to incorporate only observed interactions or also account for potential novel interactions between species that may co-occur under future conditions. This requires developing modelling frameworks capable of capturing complex feedback loops where the environment shapes species distributions, distributions determine potential interaction partners, and interactions influence species’ environmental responses.

Third, integrating network data across scales demands methodological innovation. Bridging metawebs with locally observed interaction networks while accounting for their different assumptions and limitations remains challenging. Techniques must incorporate spatial contingency and environmental context dependency into interaction network analyses across biogeographical scales. Critically, validating predictions from metaweb approaches through strategic field sampling will be essential for improving our understanding of realized interactions across gradients.

Despite these challenges, biotic interactions and biogeography have considerable potential to enhance our understanding of biodiversity patterns and their dynamics. The continued development of global ecological networks, metawebs, and standardized databases will help overcome data limitations, while advances in JSDMs and network-based approaches will improve the integration of biotic interactions into species distribution modelling. As environmental DNA sampling and automated monitoring technologies advance, new opportunities emerge to collect interaction data with greater standardization and spatial coverage, providing the empirical foundation needed to address these outstanding questions and advance predictive ecology in a rapidly changing world.

## Abbreviations

JSDMs: joint species distribution models
SDMs: species distribution models
TIB: theory of island biogeography
TTIB: trophic theory of island biogeography.
